# Acupuncture Injection Combined with Electrokinetic Injection for Polydimethylsiloxane Microfluidic Devices

**DOI:** 10.1155/2017/7495348

**Published:** 2017-02-23

**Authors:** Ji Won Ha

**Affiliations:** Department of Chemistry, University of Ulsan, 93 Daehak-Ro, Nam-Gu, Ulsan 44610, Republic of Korea

## Abstract

We recently reported acupuncture sample injection that leads to reproducible injection of nL-scale sample segments into a polydimethylsiloxane (PDMS) microchannel for microchip capillary electrophoresis. The advantages of the acupuncture injection in microchip capillary electrophoresis include capability of minimizing sample loss and voltage control hardware and capability of introducing sample plugs into any desired position of a microchannel. However, the challenge in the previous study was to achieve reproducible, pL-scale sample injections into PDMS microchannels. In the present study, we introduce an acupuncture injection technique combined with electrokinetic injection (AICEI) technique to inject pL-scale sample segments for microchip capillary electrophoresis. We carried out the capillary zone electrophoresis (CZE) separation of FITC and fluorescein, and the mixture of 10 *μ*M FITC and 10 *μ*M fluorescein was separated completely by using the AICEI method.

## 1. Introduction

Microchip capillary electrophoresis (CE) emerged in the early 1990s as an interesting and novel approach in a variety of bioanalytical applications. For example, microchip CE has been widely used for the analysis of a variety of samples that include amino acids [1], peptides [2], proteins [3], and DNA fragments [4]. Microchip CE has many advantages of decreased analysis time, integrated sample processing, high portability, high throughput, minimal reagent consumption, low analysis cost, and so forth.

In microchip CE, sample injection is one of the most critical parameters for successful separation and analysis. In this regard, it is of great importance to develop sample injection methods that lead to well-defined sample plugs for high-performance microchip CE. The electrokinetic injection is the most common method to inject a sample solution in microchip-based CE. The electrokinetic injection method is based on the electroosmotic flow (EOF) generated by applying high voltages in a microchannel. In electrokinetic injection, tee [5, 6], cross [7], and double-tee [8] injectors have been used to dispense well-defined sample plugs into a separation channel. In addition, several electrokinetic injection modes have been developed, which includes gated [9, 10], pinched [11, 12], and floating [13].

However, all electrokinetic injection modes need inconvenient voltage programs for dispensing well-defined sample plugs and require complicated hard and software systems for chip operation. Furthermore, the amounts of sample loss are quite large compared to real injected amounts into separation channel. Recently, there have been many efforts to overcome the aforementioned limitations in electrokinetic injection [14–24]. Despite the recent efforts, it is still necessary to develop simpler and more cost-effective methods for sample injection on microchips.

Very recently, we developed an acupuncture injection technique to form the well-defined sample plugs in PDMS microchannels [24]. This technique enabled us to achieve reproducible injection of 3 nL sample plug into a microchannel. Microchip CE was performed by applying a single potential in the most simplified straight channel. The advantages of this acupuncture injection in microchip CE include capability of minimizing sample consumption and voltage control hardware, capability of serial injections of different samples into a same channel, and capability of introducing sample segments into any desired position of a microchannel. However, the challenge in the previous study was to achieve reproducible, pL-scale sample injections into PDMS microchannels.

In this paper, we present an acupuncture injection combined with electrokinetic injection (AICEI) for polydimethylsiloxane (PDMS) microchips. We demonstrate that the AICEI technique allows for the injection of pL-scale segments into a PDMS microchannel while minimizing the sample loss. A pL-scale sample plug was produced by a fast-dipping method, and the sample plug was then injected into a PDMS microchip by performing acupuncture on a channel with a homemade capillary needle and applying a high voltage to drive the sample plug.

## 2. Experimental Section

### 2.1. Chemicals and Materials

PDMS was purchased from Dow Corning (Midland, MI, USA) under the product name Sylgard 184. Microslides were purchased from VWR Scientific Inc. (West Chester, PA, USA). All reagents used in this paper were of analytical grade and were obtained from Sigma-Aldrich (St. Louis, MO, USA). All aqueous solutions were prepared by using water purified with a Milli-Q purifying system (Millipore, Milford, MA, USA). An injector composed of a micrometer, a syringe holder, and a translator was a part of a contact angle meter (MODEL: KRUSS G10) and was purchased from Marktech Co. (Seoul, Republic of Korea). 30 gauge-disposable needles were obtained from Becton Dickinson Co. (Franklin Lakes, NJ, USA).

### 2.2. Fabrication of PDMS Microchips

PDMS microchips in this paper were fabricated by photolithography replica molding method [25–28]. Channel networks were designed by using a computer aided design software package (AutoCAD; Autodesk, San Rafael, CA, USA). The network designs were converted into a sheet of mask. A 25 *μ*m thick film of a negative photoresist (SU-8 50; MicroChem, Newton, MA, USA) was spin-coated on a silicon wafer of 100 mm diameter (LG Siltron, South Korea), exposed to UV light through the mask, and then developed in propylene glycol methyl ether acetate (Aldrich, Milwaukee, WI, USA) to reveal a master with a positive relief pattern of the photoresist. The master was silanized by placing it in a vacuum desiccator for 2 h along with a vial containing a few drops of a silanizing agent, tricholoro(3,3,3-trifluoroproyl)silane (Aldrich). A 10 : 1 mixture of PDMS oligomer and cross-linking agent (Sylgard 184) was poured onto the master and then degassed under vacuum. After at least 3 h of curing at 75°C, a PDMS replica was peeled from the master to yield the negative relief of channel networks. The reservoirs were defined by punching holes at the dead ends of channels. A flat slab of PDMS was made similarly by casting the Sylgard 184 mixture against a silanized silicon wafer. A PDMS replica and a flat slab were rinsed in methanol and dried under a stream of nitrogen. The surfaces of the two PDMS pieces, which will face each other, were oxidized by corona discharge generated from a Tesla coil (BD-10A; Electro Technic Products, Chicago, IL, USA) for 2 min. Immediately after the treatment, the two pieces were brought into conformal contact, and a complete irreversible seal was accomplished spontaneously in 3 h. Figure S1 (in Supplementary Material available online at https://doi.org/10.1155/2017/7495348) shows a photographic image of PDMS microchips used for the CZE experiment in this study. The channel length from reservoir 1 to reservoir 2 was 38 mm, while the channel width and height was 100 *μ*m.

### 2.3. Fabrication of a Capillary Needle

**Figure 1:**
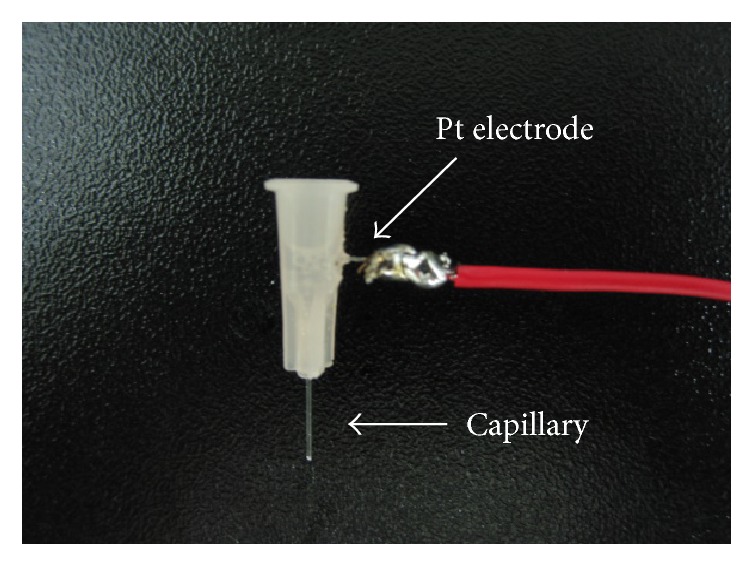
A photographic image of a homemade capillary needle designed for AICEI method. The tip was grinded into a slant face. Pt electrode was inserted and firmly fixed into the cone of a capillary needle.

In the present study, we employed a homemade capillary needle for electrically driven acupuncture injection (Figure 1). The outer and inner diameters of the capillary needle were 300 *μ*m and 100 *μ*m, respectively. In this sample injection method, the plastic cone of a capillary needle serves as a reservoir where a buffer solution is contained. Therefore, a Pt electrode was inserted into the plastic cone and was firmly fixed with an adhesive. The tip of a capillary needle was modified and grinded into a slant face.

### 2.4. Apparatus and Instrumentation

Electrokinetic injection and separation were controlled by using a computer-controlled high-voltage supplying system equipped with a high-voltage power supply (MP5; Spellman High Voltage Electronics, Plainview, NY, USA), a high-voltage relay (K45C332; Kilovac, Santa Barbara, CA, USA), and a homemade voltage dividing system. A LabVIEW (National Instruments, Austin, TX, USA) program written in-house and a multifunction I/O board (Lab-PC-1200; National Instruments) were used for instrument control and data acquisition.

For laser-induced fluorescence (LIF) detection, a laser beam was incident on the chip at an angle of 45° and focused on near the end of the separation channel with a planoconvex fused-silica lens (100 mm focal length; CVI Laser Optics & Coatings, Alibuquerque, NM, USA). The fluorescence light was collected from below the chip with a 10x microscope objective (numerical aperture, 0.3; Nikon, Japan) and directed to a photomultiplier tube (HC120-01; Hamamatsu, Bridgewater, NJ, USA) through a 0.5 mm pinhole located at the image plane of the objective lens. Band pass filters (10 nm bandwidth; CVI Laser Optics & Coatings) were used for eliminating the scatter of the excitation line of the argon ion laser (Lexel 95; Lexel Laser, Fremont, CA, USA).

### 2.5. Capillary Zone Electrophoresis

Before performing the CZE experiment, the channel networks were washed with methanol and deionized water for 10 min. A 4 mM boric acid and 20 mM Tris(hydroxymethyl) aminomethane buffer (TRIS) mixture (pH 9.0) containing 10 mM sodium dodecyl sulfate (SDS) was used as electrophoretic medium. A mixture of 10 *μ*M FITC and 10 *μ*M fluorescein was used as the sample. CZE separation was performed using the straight channel microchip shown in Figure S1. In this method, only a single potential was applied at two reservoirs for CZE separation. In this study, sample was detected by LIF detection method in which an argon ion laser (Lexel 95; Lexel Laser, Fremont, CA, USA) was employed as the excitation source.

## 3. Results and Discussion

The generation of well-defined sample plugs is critical for high-performance CE separation and analysis. In this study, the sampling was carried out by a fast-dipping method based on a capillary action. More specifically, the capillary needle filled with a buffer solution (50 mM phosphate) was sequentially dipped into perfluorodecalin and sample solutions in order to make a sample segment inside a needle (Figure S2). The reason for using perfluorodecalin immiscible with aqueous phase is to avoid the diffusion and dilution of sample segments to be injected into a separation channel. Figure S3 shows the effect of perfluorodecalin on the diffusion of a sample plug. Figure S3A is a CCD image of a sample plug with perfluorodecalin at both ends of the sample plug. We observed that the diffusion of a sample segment into the buffer solution was avoided in the presence of perfluorodecalin segments. However, the dilution was observed in the absence of perfluorodecalin at both ends of the sample plug. Therefore, this dipping method allowed us to produce a well-defined aqueous sample plug in between perfluorodecalin segments inside the capillary needle.

**Figure 2:**
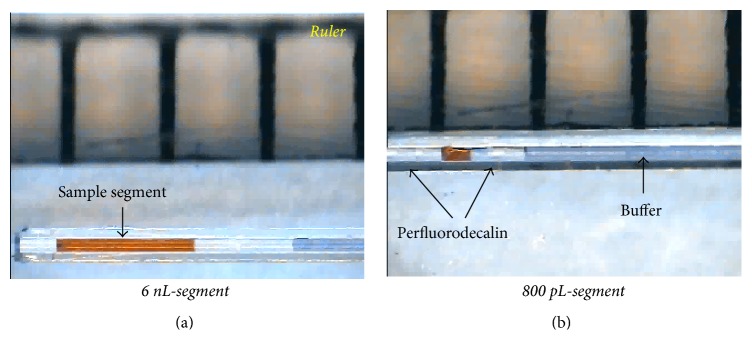
(a) CCD image of a sample segment (red-color) in between perfluorodecalin plugs. The loaded volume was calculated to be ~6 nL. A buffer solution was mixed with blue-ink for clearer demonstration. (b) CCD image of a sample segment (red-color) with the loaded volume of 800 pL.

As a next step, it is important to determine the amounts of sample loaded into the capillary needle by the fast-dipping method. In this study, the length of a sample plug was measured (or estimated) by using a ruler, and then we determined the loaded volume by calculating the volume of a cylinder.  Figure 2 shows sample segments (red-colour) loaded into the capillary needle. In Figure 2(a), the loaded volume was calculated to be about 6 nL. We further tried to reduce the volume of a sample plug loaded into the capillary needle by the fast-dipping method, and we could push it down to 800 pL as shown in Figure 2(b). This indicates that pL-scale sample segments can be generated in the capillary needle by the fast-dipping method. Therefore, it should be noted that we developed a new way to form well-defined, pL-scale sample segments in a capillary needle without a diffusion problem.

**Figure 3:**
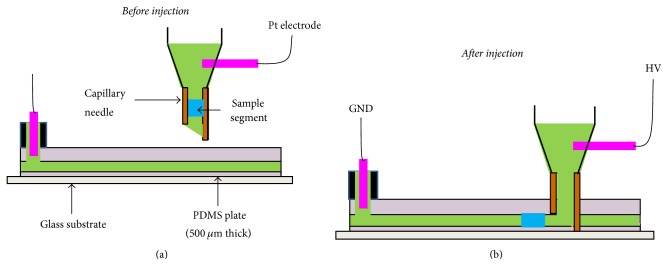
Schematic diagram to show the AICEI technique. (a) Before injection: a homemade capillary needle position is adjusted in the horizontal plane. (b) After injection: a sample segment is injected by applying acupuncture on a channel and applying a high voltage (single potential, 1 kV).

The next crucial step is to dispense the sample plug into a PDMS microchip. Our idea is to introduce the sample plug by applying acupuncture on a microchannel and applying a high voltage for inducing EOF. More concretely, Figure 3 depicts a schematic of AICEI method. Initially, the homemade capillary needle position is adjusted in the horizontal plane with the assistance of a CCD camera (Figure 3(a)). Then, the PDMS microchannel is carefully acupunctured vertically with the capillary needle (Figure 3(b)). After applying acupuncture, the sample plug is introduced into a separation channel by applying a high voltage to generate EOF as shown in Figure 3(b).

**Figure 4:**
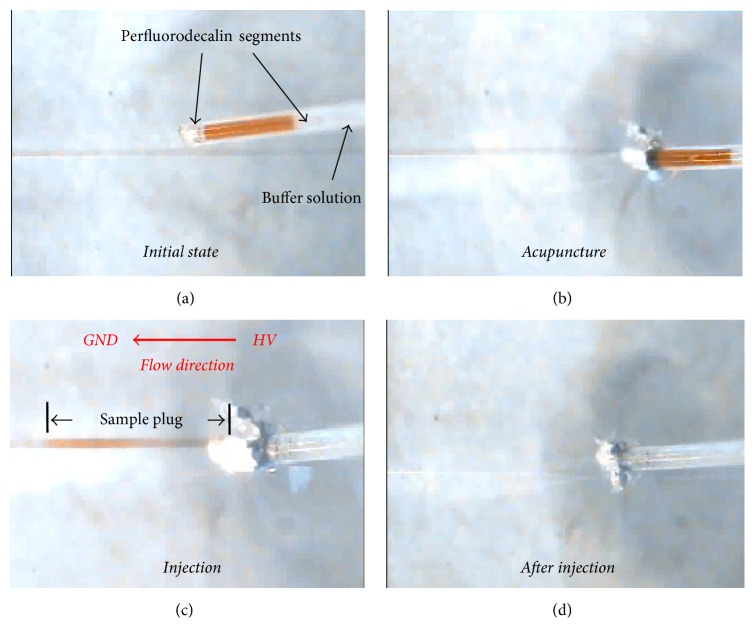
Consecutive CCD images recorded at the different stages of sample injection using the AICEI method from the top view. (a) Initial state, (b) acupuncture, (c) sample injection, and (d) after injection.

Now, it is important to experimentally verify the approach described in Figure 3. In the present study, we developed an instrumental setup to realize the AICEI method (Figure S4). The instrument consists of a high voltage supplier, a CCD camera, a chip holder, and an injector. The injector, a part of a contact angle meter, is composed of a micrometer, a syringe holder, and a translator (Figure S4B). After setting up the system, a microsyringe with a sample plug in the capillary needle was fixed to the holder of injector. We then tried to inject sample segments into a PDMS microfluidic channel by the AICEI method in Figure 3.  Figure 4 shows consecutive CCD images of introducing a sample plug using the AICEI method from the top view. Initially, the needle position in the horizontal plane was adjusted with the assistance of a CCD camera (Figure 4(a)). We then inserted the capillary needle into the microchannel until it touches at the surface of the glass substrate (Figure 4(b)). After this, we applied a high voltage (1 kV) to drive the red-coloured sample plug into the microchannel (Figure 4(c)). Finally, a sample plug was flowed in the microchannel (Figure 4(d)). At the end of experiment, the capillary needle was pulled out carefully from the microchip. Therefore, we experimentally confirmed that the AICEI technique can be used to inject well-defined sample plugs into a PDMS microchip. Furthermore, this AICEI method allows us to achieve pL-scale sample injection, which was not achieved by the acupuncture injection in our previous study [24].

**Figure 5:**
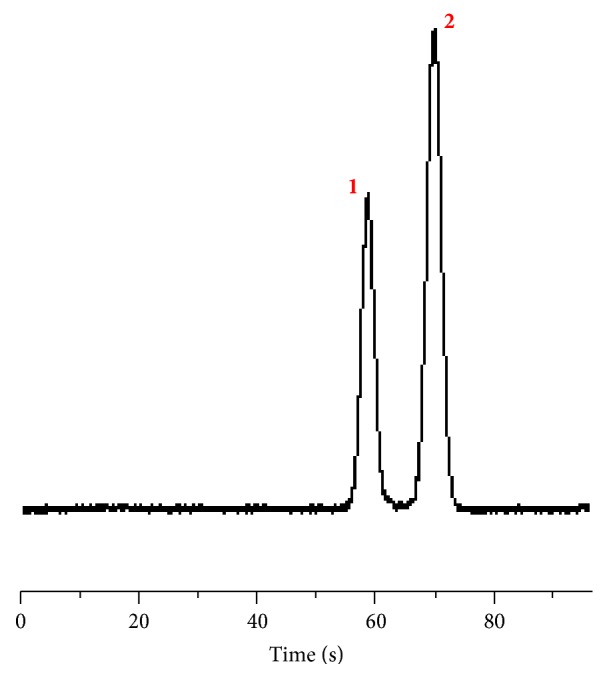
CZE separation of 10 *μ*M FITC (peak 1) and 10 *μ*M fluorescein (peak 2) using the AICEI method.

Last, it is necessary to demonstrate that the AICEI method can be directly used for the microchip CE separation. We therefore performed CZE separation of FITC and fluorescein by using the AICEI method. As shown in the electropherogram (Figure 5), the mixture of 10 *μ*M FITC and 10 *μ*M fluorescein was separated completely by the AICEI method. Compared to typical gated and pinched mode sample injection, this AICEI method requires only a single potential application, which is greater advantage. Furthermore, the AICEI method provides more advantages including capability of minimizing the sample loss, capability of injecting samples into any position of a channel, and ease in fabricating microchips.

## 4. Conclusions

In summary, a new acupuncture injection technique in combination with electrokinetic injection is introduced for polydimethylsiloxane (PDMS) microfluidic devices. This acupuncture injection technique has several advantages that include capability of minimizing sample consumption and voltage control hardware, capability of serial injections of different sample solutions into a same microchannel, and capability of introducing sample segments into any position of a microchannel. In this work, we present that pL-scale sample plugs can be injected into a PDMS microfluidic device by performing acupuncture on a channel with a homemade capillary needle and applying a high voltage to drive the sample plugs for microchip CE separation ad analysis. Furthermore, we demonstrate that the AICEI method can be used for CZE separation in the most simplified straight channel with a single potential.

## Supplementary Material

Figure S1: A photographic image of PDMS microchip.
Figure S2: A schematic to show a fast-dipping method for generating a sample segment in the capillary needle via a capillary action.
Figure S3: The effect of perfluorodecalin on the diffusion of a sample plug.
Figure S4: Photographic images to show the experimental setup for the electrically-driven acupuncture injection method.
