# Supplementary material for: Acupuncture Injection Combined with Electrokinetic Injection for Polydimethylsiloxane Microfluidic Devices
Source: J Anal Methods Chem. 2017 Feb 23;2017:7495348. doi: 10.1155/2017/7495348 (PMC5343277; doi:10.1155/2017/7495348)
Supplement: Supplementary file 1 — Figure S1: A photographic image of PDMS microchip. Figure S2: A schematic to show a fast-dipping method for generating a sample segment in the capillary needle via a capillary action. Figure S3: The effect of perfluorodecalin on the diffusion of a sample plug. Figure S4: Photographic images to show the experimental setup for the electrically-driven acupuncture injection method. [file 7495348.f1.doc]

**Supplementary Information**

**Acupuncture Injection Combined with Electrokinetic Injection for Polydimethylsiloxane(PDMS) Microfluidic Devices**

Ji Won Ha^*^

Department of Chemistry, University of Ulsan, 93 Daehak-Ro, Nam-Gu, Ulsan, 44610, South Korea

^*^To whom correspondence should be addressed.

**J. W. Ha**

E-mail: jwha77@ulsan.ac.kr

This document contains experimental methods and additional supporting figures (Figure S1 to S4).

**Supplementary Figures**


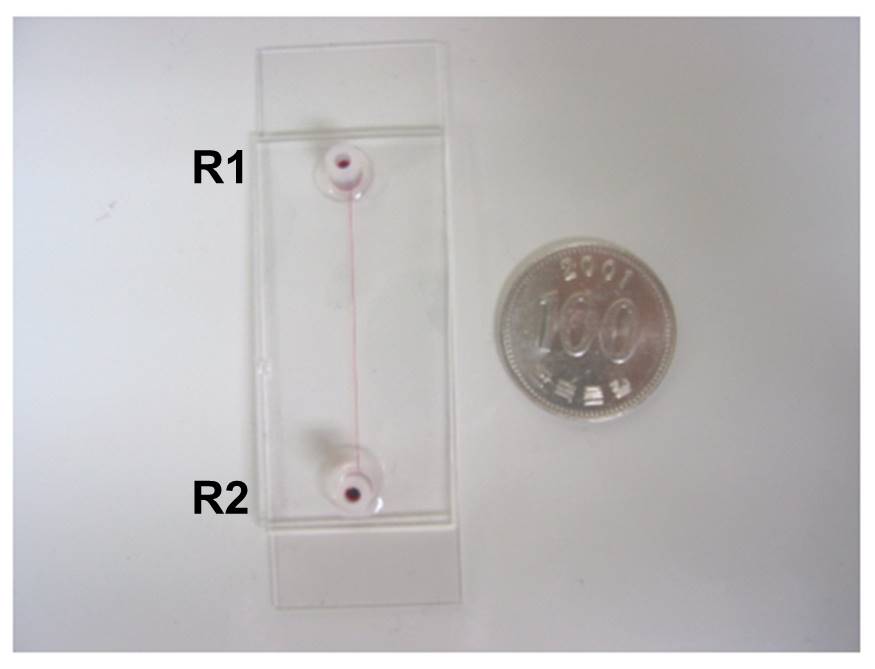


Figure S1: A photographic image of PDMS microchip with a straight channel (Width: 100 µm, Depth: 100 µm). The length from R1 to R2 was 38 mm. An Ink solution was flowed to show a microchannel clearly.

**
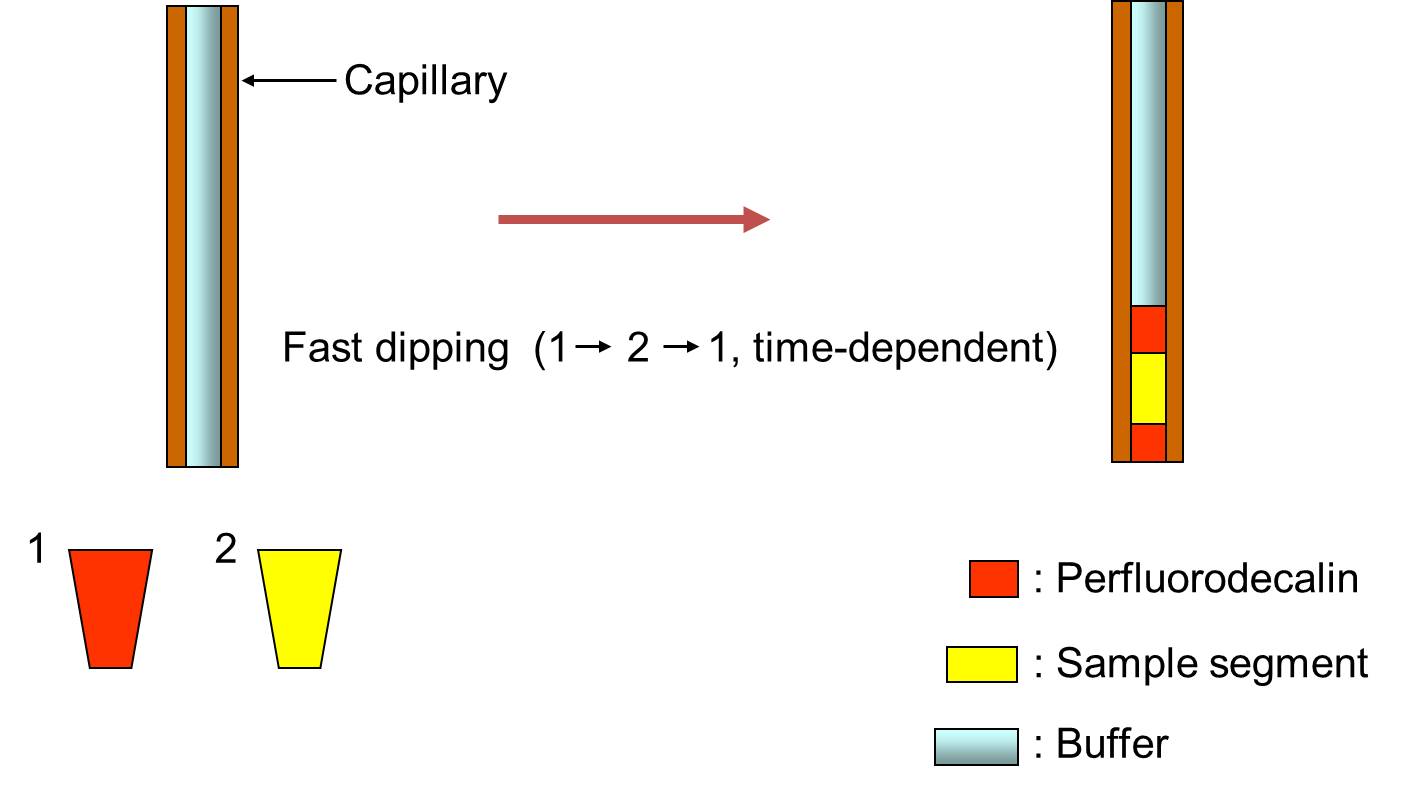
**

Figure S2: A schematic to show a fast-dipping method for generating a sample segment in the capillary needle via a capillary action. The needle was sequentially dipped into perfluorodecalin and sample solutions. This results in a nL-scale sample segment in between perfluorodecalin segments.

**
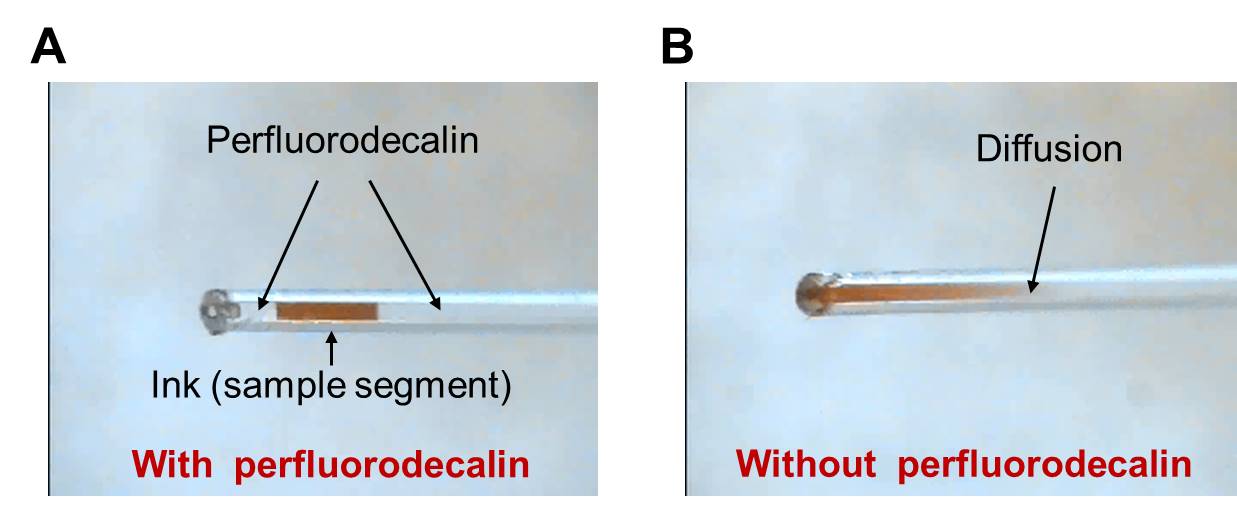
**

Figure S3: The effect of perfluorodecalin on the diffusion of a sample plug. (A) CCD image of a sample plug with perfluorodecalin at both ends of the plug. The diffusion or dilution was avoided in the presence of perfluorodecalin. (B) CCD image of a sample plug without perfluorodecaline segments. The dilution was observed in the absence of perfluorodecalin.

**
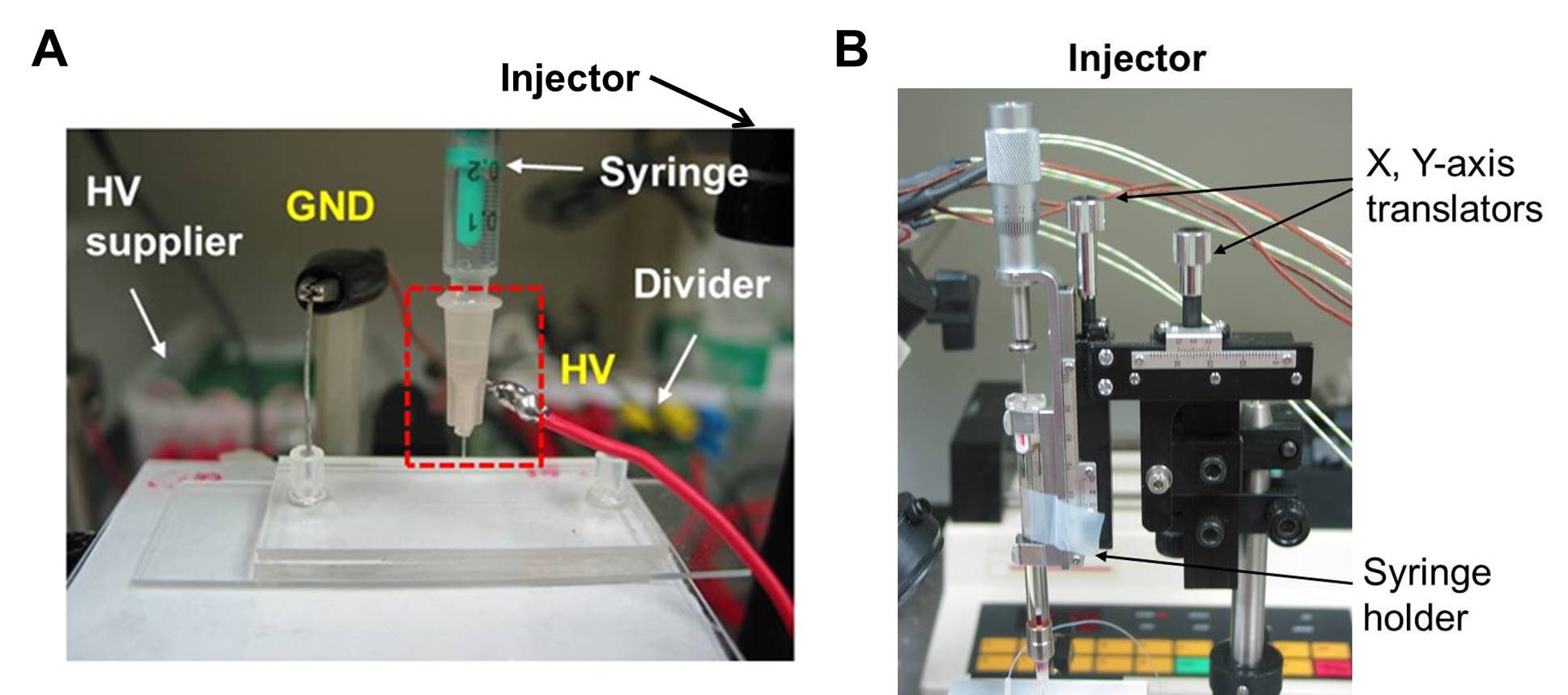
**

Figure S4: (A) A photographic image to show the experimental setup for the electrically-driven acupuncture injection method. (B) A magnified view of an injector where a micro-syringe is fixed.
